# Diatomaceous Earth—Lightweight Pozzolanic Admixtures for Repair Mortars—Complex Chemical and Physical Assessment

**DOI:** 10.3390/ma15196881

**Published:** 2022-10-03

**Authors:** Milena Pavlíková, Pavla Rovnaníková, Martina Záleská, Zbyšek Pavlík

**Affiliations:** 1 Department of Materials Engineering and Chemistry, Faculty of Civil Engineering, Czech Technical University in Prague, Thákurova 7, 166 29 Prague, Czech Republic; 2 Institute of Chemistry, Faculty of Civil Engineering, Brno University of Technology, Žižkova 17, Veveří, 602 00 Brno, Czech Republic

**Keywords:** diatomaceous earth, pozzolanic activity, Portland cement substitution, heat release, rheology, strength activity index, improvement of mechanical parameters

## Abstract

The presented research is focused on the complex assessment of three different types of diatomaceous earth and evaluation of their ability for application as pozzolana active admixtures applicable in the concrete industry and the production of repair mortars applicable for historical masonry. The comprehensive experimental campaign comprised chemical, mineralogical, microstructural, and physical testing of raw materials, followed by the analyses and characterization of pozzolanic activity, rheology and heat evolution of fresh blended pastes, and testing of macrostructural and mechanical parameters of the hardened 28-days and 90-days samples. The obtained results gave evidence of the different behavior of researched diatomaceous earth when mixed with water and Portland cement. The differences in heat evolution, initial and final setting time, porosity, density, and mechanical parameters were identified based on chemical and phase composition, particle size, specific surface, and morphology of diatomaceous particles. Nevertheless, the researched mineral admixtures yielded a high strength activity index (92.9% to 113.6%), evinced their pozzolanic activity. Three fundamental factors were identified that affect diatomaceous earth’s contribution to the mechanical strength of cement blends. These are the filler effect, the pertinent acceleration of OPC hydration, and the pozzolanic reaction of diatomite with Portland cement hydrates. The optimum replacement level of ordinary Portland cement by diatomaceous earth to give maximum long-term strength enhancement is about 10 wt.%., but it might be further enhanced based on the properties of pozzolan.

## 1. Introduction

In the production of repair mortars, materials compatible with those initially inbuilt should find helpful. However, today’s building practice applies to the renewal of masonry repair mortars based mostly on cement, hydrophobic agent, modifying polymer-origin additives, and different fillers. Based on our previous research on mortars for the restoration of historical buildings [1], pure cement mortar should be avoided due to their incompatible porosity, mechanical strength, rigidity, and low water vapor permeability [2]. Therefore, lime-, natural hydraulic lime-, and blended cement-based mortars have been developed within the last decade and are increasingly applied in reconstruction treatments [3,4]. 

Together with compatibility and functionality issues, there is a rising interest in producing composite cement due to increasing ecological awareness and diminishing natural resources [5]. As global manufacturing of Portland cement contributes to approx. 7% of the total anthropogenic CO_2_ emissions, the mounting attention on environmental aspects of material conversion has influenced research towards possible modifications of Portland cement to meet better demands for sustainability in the construction sector [6]. This is done by using different types of low-carbon additives and admixtures and changing the composition and, thus, the performance of cement [7,8]. It is usually replaced in composite cement as part of clinker by supplementary cementitious materials (SCMs) [9,10]. They can be used by blending Portland cement at the cement plant or replacing Portland cement at the concrete plant. In most cases, the SCM can improve the durability and mechanical properties of conventional binders, especially if SCM are pozzolans, siliceous, or silico-aluminous mineral admixtures. The pozzolanic reaction involves two main parameters: the maximum calcium hydroxide consumption and the rate of hydration. These parameters depend mainly on the nature of pozzolanic material, its chemical and mineralogical composition, and its particle size [11].

The most widespread used SCMs have been fuel ash [12,13,14] and ground granulated blast furnace slag [15,16,17]. Metakaolin [18,19,20] was also applied to reduce the amount of cement produced and thus bring environmental and economic benefits. However, the availability of these materials becomes increasingly problematic, which will be further worsened in the near future. For this reason, there is a need to find and assess other alternatives to reduce Portland cement production and its harmful environmental impact [21]. 

Considering the necessity to find alternatives to well-established SCMs, three types of diatomaceous earth were investigated and assessed as prospective partial cement substitutes in a blended binder for repair mortars. In the case of pyramid stones in Egypt, it is believed that the fine limestone particles were bound together with calcium silicates produced from a reaction between diatomaceous earth and lime [22]. Diatomaceous earth deposits were found in Fayoum, roughly 70 km south of Cairo, Egypt [23].

The diatomaceous earth or diatomite is a lightweight rock of sedimentary origin, mainly consisting of the fossilized skeletal remains of diatom, a unicellular aquatic plant related to the algae skeleton fragments of diatom algae (Diatomea and Radiolaria), called frustules, with a wide variety of shape and size, which ranges from 0.75 µm to 1500 µm [24]. It is a highly porous structure containing up to 80–90% voids, constituting mainly amorphous silica with minor amounts of alumina and ferric oxide, followed by crystalline quartz, muscovite, feldspar [25], and characterized by a specific surface area up to 200 m^2^ [26]. 

The low cost, abundance, easy availability, excellent biocompatibility, non-toxicity, thermal stability, and chemical inertness make diatomite an intriguing material for applications ranging from filtration to pharmaceutics [27,28]. The unique combination of physical and chemical properties of diatomite makes it applicable for several industrial uses, as a filtration media for chemicals, various beverages, drinking water, sugar, etc., without altering their natural properties or for the removal of inorganic and organic pollutants. Because of its high porosity, diatomaceous earth is used not only as a beverage filter material but also as a mildly abrasive or mechanical pesticide. Usually, diatomite mined from geological deposits must be purified before being used; thermal pre-calcination and HCl washing are the treatments generally used to increase powder quality and to make the biomaterial inert as filter support [29]. In the construction industry, diatomite finds use in lightweight brick manufacturing [30,31] to produce ceramics [32,33,34] for the improvement of MOC composites [35] and as a part of shape-stabilized phase change materials [36,37]. It is also applied in designing and developing high-temperature resistant and thermal insulation materials [38,39] and as lightweight aggregate manufacturing mortar and concrete [40,41].

Different types of mineral admixtures are added to increase the cementitious material content, improve the workability of harsh cement blends, and achieve high strength without causing high internal temperatures. Aruntas and Tokyay [42] indicated that adding diatomite in Portland cement significantly affects grain size, i.e., increasing fineness. When diatomaceous earth is blended with ordinary Portland cement, it undergoes strength increases or simply acts as a filler while maintaining similar strength development [43,44]. Kastis [45] reported on the pozzolanic activity of diatomite, leading to the formation of higher amounts of hydrated products. Similarly, dos Santos and Cordeiro [46] observed enhancement of the pozzolanic activity of diatomite by grinding, i.e., by an increase of specific surface area. On the other hand, Aydin and Gül [47] indicated that the rise in diatomite content in concrete resulted in a sudden drop in its compressive strength. Due to the high specific surface area, ordinary Portland cement blended with diatomaceous earth requires more water, leading to inferior concretes [48,49]. Similarly, Lemons [26] reported that the high porosity of diatomite leads to increased water demand, limiting the substitution level of untreated diatomite earth [50,51]. As the use of diatomite as SCM brings some apparent advantages, and also drawbacks, there is a need to extend the present state of knowledge in the field of diatomaceous earth application in Portland cement blends and to conduct additional, comprehensive analyses of their effect on the final properties of the hardened products. In relation to high amorphous silica content, diatomite is assumed to be an environmentally friendly and cost-effective SCM. The up to now reported data on the application of diatomaceous earth as pozzolans are often contradictory, pointing out some adverse effects on the performance and properties of diatomite-cement blends. Therefore, research on the effectiveness of reactive SiO_2_-rich materials is highly actual and necessary to contribute to developing less energy-demand blended binders and, thus, construction materials. Moreover, the diatomaceous earth must be studied case by case due to its variation in mineralogical and chemical composition, morphology, fineness, pre-processing, etc. In this respect, the objective of the presented work was to investigate diatomite-Portland cement blends in the form of pastes with a constant water/binder ratio. Three types of diatomaceous earth were incorporated in cement paste mixtures as SCMs. The experimental campaign comprised the characterization of raw materials, behavior, and properties of fresh and hardened blended cement pastes. A broad set of experimental techniques was used to analyze the effects of material conversion in the diatomite-Portland cement system. Laboratory tests and measurements showed that the diatomite samples exhibited good pozzolanic activity and could substitute the currently used natural pozzolans. The best compressive strength values at later ages were obtained using the diatomaceous earth with the highest amorphous silica content. The late compressive strength of most pastes modified with diatomite was improved compared to that of reference cement paste which agrees with previously published reports [43,51,52]. 

## 2. Materials and Methods

### 2.1. Raw Materials

CEM I 42.5 R produced in the cement factory in Radotín, Czech Republic, was used as a primary binder. This hydraulic binder is made in accordance with the standard EN 197-1 [53]. The diatomaceous earth used in prepared pastes, denoted as BOR, is quarried in the Miocene Mydlovary formation in the South Bohemian basins. There is located at the biggest quarry in the Třeboň (Czech Republic) basin in the region between Ledenice and Borovany. The diatomaceous earth quarried here is pale-colored, grey to ochre, earthy or loosely cemented, and placed nearly horizontally. In some upper parts of this diatomite location, the spongo–diatomite layer is present. In the middle part of the quarry, the diatomite layer achieved about 100 m of thickness, whereas the average thickness is about 5.85 m. During sedimentation processes of Mydlovary formation, several ingressions of the sea onto land took place. The Borovany profile was opened in 1909 and belonged to the Schwarzenberg demesne. Diatomite mining continued after World War Two, and the new fabric named Colofrig was discovered in 1961. The tested diatomite (commercially labeled F4) is produced in LB MINERALS, s.r.o.(Horní Bříza, Czech Republic), a member of the LASSELSBERGER group, as a powder useful for filtration processes. For the comparison, two different types of calcined diatomaceous earth originating from Italy (Dal Cin Gildo S. p. a., Concorezzo, Italy), denoted as ENO3 and ENO7 (commercially Enorandall^TM^ 3 and Enorandall^TM^ 7), were tested. 

### 2.2. Chemical and Physical Analysis of Raw Materials

The ability of SCMs to take part in the hydration reaction of blended binders depends on their chemical and physical properties. The total chemical composition of tested raw materials was determined using classical chemical analysis according to the Czech standard ČSN EN ISO 11885 and ČSN 720101. In the first step, the analyzed materials were melted together with LiBO_2_ and then dissolved in the solution. The solution composition was analyzed with ICP-OES (Inductively Coupled Plasma Atomic Emission Spectroscope) Jobin Yvon ULTIMA 2, Horiba Scientific. The total chemical composition of examined raw materials is presented in Table 1.

The content of four main clinker phases in PC calculated based on Bogue’s equations [54] was the following: C_3_S 65.9 wt.%, C_2_S 4.7 wt.%, C_3_A 7.4 wt.%, and C_4_AF 7.3 wt.%, respectively. The differences in the chemical composition in the case of diatomaceous earth are caused due to their origin. The powders ENO3 and ENO7 are of seawater origin, and the diatomite BOR has a freshwater origin, see the mass content of alkalis in Table 1.

The complete chemical analysis does not say anything about the reactivity of tested diatomaceous earth in an alkaline environment. In that case, there is essential to know the content of reactive amorphous phases. According to the standard ASTM C618-91 [55], the total content of the hydraulic oxides (SiO_2_ + Al_2_O_3_ + Fe_2_O_3_) in pozzolana active materials is recommended at 70 wt.% and the content of active SiO_2_ no less than 25 wt.%. For that reason, the XRD (X-Ray Diffraction) analysis of diatomite samples was performed. It was done using the Siemens D-5000 X-Ray diffractometer with Bragg-Brentano configuration (CuKα source, λ = 1.5405 Å). The phase identification was performed with Crystallographica Search Match (Version 2.0.3.1, Oxford Cryosystems, Oxford, UK) according to the Whole Pattern Fitting Structure Refinement invented by Rietveld [56]. The results of the XRD analysis are introduced in Table 2.

The differences in mineral and phase composition of the examined minerals are visible. The amount of amorphous was the highest for ENO7. The other two materials have about 43.0% (ENO3) and 22.6% (BOR) lower content in the amorphous phase. The presence of cristobalite, which anneals at high temperatures, declares the method of raw material processing. While diatomite BOR is produced only mechanically, the other two, ENO3 and ENO7, are calcination processed. The opal content in the sample ENO3 indicates the compacting rate because opal is replaced with quartz in compacted rocks. The presence of kaolinite and illite in the case of diatomite BOR is adequate for the quarry composition.

**Figure 1:**
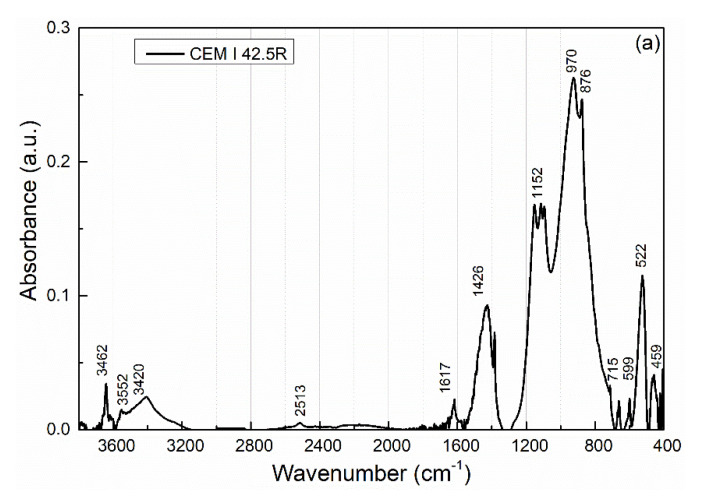

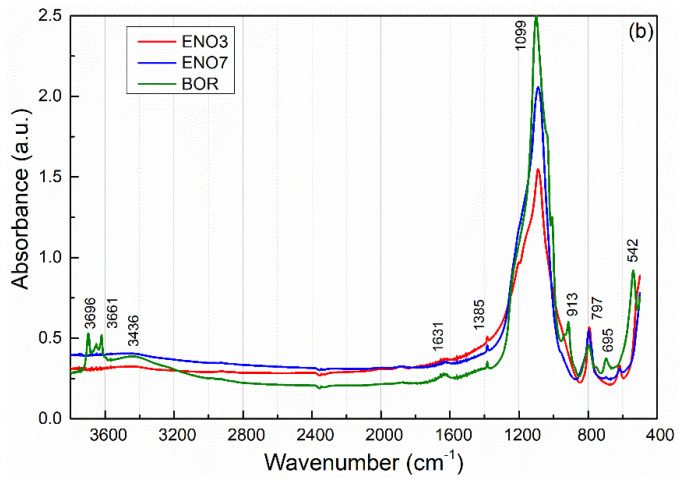
FTIR spectra of raw materials: (**a**) CEM I 42.5R, (**b**) diatomaceous earth.

The chemical composition of raw materials was also examined with IR (infrared) analysis (see Figure 1a,b). Infrared spectra were measured using FTIR spectrometer Nicolet 6700 (Thermo Scientific, Waltham, MA, USA). Spectra were collected after 32 scans at 4 cm^−1^ resolution in absorbance mode. The samples were prepared by the standard KBr pellets method, the sample powders were dispersed in KBr (1/150 mg), and the transparent pellets were tested with a transmission accessory. The spectral regions were normalized to enable comparison of individual samples. Peak deconvolution using Gaussian peak fitting was performed to evidence the stretching mode contribution of the different functional groups. 

Table 3 and Table 4 show the assignment of the major absorption bands of the tested raw materials.

FTIR spectra of siliceous materials exhibit peaks in two distinct regions [61]: peaks at >2500 cm^−1^ and <1300 cm^−1^. The first region is typical for O-H stretching vibration of absorbed or molecular water, while the second occurs due to several silica modes. The peak at 400–500 cm^−1^ can be assigned to the rocking motion of oxygen atoms bridging silicon atoms in siloxane bonds Si-O-Si. The symmetric vibrations of silicon atoms in siloxane bonds occur at ~800 cm^−1^. The most prominent peak in the spectrum presented at ~1100 cm^−1^ is dominated by the antisymmetric motion of silicon atoms in siloxane bonds. It consists of three components: a broad shoulder centered at 1115–1130 cm^−1^ assigned to longitudinal optical mode (LO ν_as_ -Si-O-Si), a strong peak centered at 1035–1045 cm^−1^ (transverse optical mode TO ν_as_ -Si-O-Si) and weak absorption at 950 cm^−1^ due to silanol (Si-OH) stretching vibrations [62].

**Figure 2:**
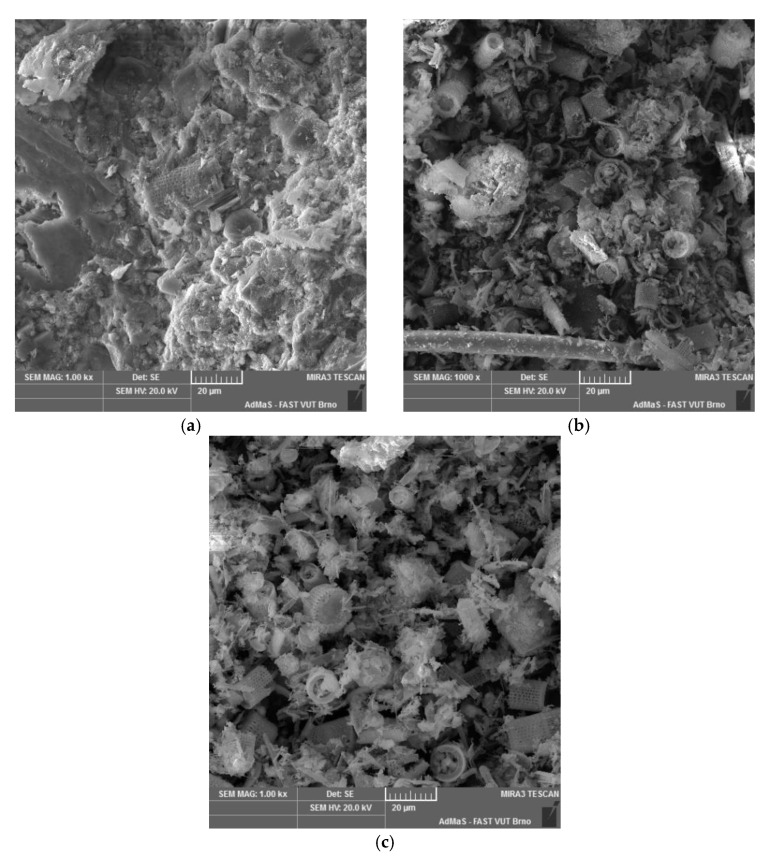
SEM analysis of diatomaceous earth (**a**) ENO3, (**b**) ENO7, (**c**) BOR.

The microstructure and morphology of diatomaceous earth particles are well apparent in Figure 2. The microstructural analysis was conducted using a scanning electron microscope (SEM) JSM-840, JEOL, Ltd., Equipped with a LaB6 cathode, thus providing a very high resolution. 

Also visible, the sedimentary rock called diatomite or diatomaceous earth is composed of fossil SiO2-based cells of dead organisms called Diatomacea. In our case, all samples shove the centric genera [63]. These organisms have properties of both plants and animals, and for that reason, they create the fifth alone kingdom of Eukaryotes named Chromista [64]. The oldest fossils of Diatomacea come from the older Cretaceous period of Mesozoic, i.e., they date back to 120 million years ago. About 11 million cells form 1 cm^3^ of diatomaceous earth. The frustules are the hard and porous cell wall or external layer of Diatomacea, composed almost purely of polymerized SiO_2_. They are coated with a layer of organic substance, the acid polysaccharide Diatotepin [65]. The double-layered cell grows due to the active catching of siliceous acid from the environment with the speed of 4–18 molecules per second [66].

Physical parameters tested characterize raw materials from the view of size, shape, distribution, and especially specific surface of particles. The specific density of analyzed powders was also researched. 

**Figure 3:**
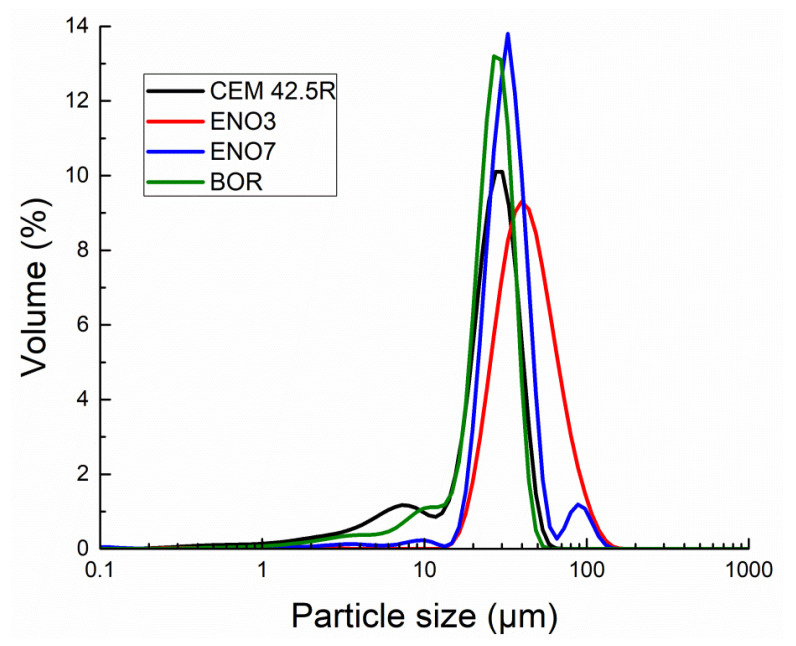
Particle size distribution of diatomaceous earth and CEMI 42.5R.

The particle size distribution of cement and diatomaceous earth was determined by a laser diffraction working device, Analysette 22 Micro Tec plus, Fritsch. The calculated values of particle volume fraction 10, 50, and 90% corresponding to the particle size are presented in Table 5. The particle size distribution curves of tested diatomaceous earth compared to PC CEMI 42.5 R are introduced in Figure 3. 

A comparison with cement CEM I 42.5 R (the main peak at the particle size distribution curve was recorded for a pore diameter of 30.2 µm) showed that the powders of tested diatomaceous particles in the case of ENO 3 and ENO7 were coarser, nominally 39.8 µm and 32.7 µm, respectively. On the other side, the sample labeled BOR is slightly finer (26.8 µm). However, their particle size is sufficient for the intended application of the analyzed diatomaceous earth as a partial cement replacement. 

Another parameter for describing the fineness of the tested materials is the specific surface area because most of the surface area comes from the smallest particles. The most common method for characterizing the surface area is the Blaine air permeability test for cement testing described in EN 196-6 [67]. The test is based on the fact that the rate at which air can pass through a porous bed of particles under a given pressure gradient is a function of the surface area of the powder. The metal chamber of known cross-sectional area and volume is filled with a known mass of fine powder, and then the time required to pass a known volume of air through the powder is measured. The surface area is determined through an empirical equation developed by measuring a standard, the powder of a known surface area, using the same instrument. The resulting values, called the Blaine fineness, are presented in Table 6. 

The other way to explore the specific surface area is a measurement based on physisorption (physical adsorption). Applying inert gases, such as nitrogen, enables the determination of specific surface, total pore surface, total pore volume, and pore size distribution. For the measurement, apparatus Sorptomatic 1990 (Thermo Scientific, Waltham, MA, USA) was used. The equipment consists of two main parts: control and analytical sections. Common coolant for the analyses is liquid nitrogen (−192 °C); different ovens are available to perform analyses from room temperature up to 450 °C. The measured data were calculated according to the BET theory to obtain the values in Table 6.

As other fundamental physical characteristics of each material were tested, also specific density was experimentally determined using a fully automatic helium pycnometer Pycnomatic ATC (POROTEC). The instrument works under isothermal conditions, monitored with Peltier’s thermocouples, and the analytical process is controlled with a microprocessor. The tested sample’s maximum volume up to 62 cm^3^ guarantees precise density determination, which is presented in Table 6.

The reactivity of cement and pozzolana active materials is related to their Blaine fineness and specific surface area. In this case, the explored diatomaceous earth exhibit much higher Blaine fineness than measured for PC. For ENO3, ENO7, and BOR, the Blaine fineness values increased by 118%, 418%, and 482%, respectively. It is generally accepted strength of PC increases with its fineness [68]. It means the activity of mineral admixtures might also be enhanced by higher Blaine fineness. Similarly, mineral admixture, which is softer than PC in given fineness, results in improved durability of blended mortar [69].

The specific surface area is a better parameter for describing the fineness of powder materials. Still, it is difficult to pin down because most materials have features at different lengths, so the more sensitive the measurement, the more surface area will be found. Different measuring techniques thus give different values. As Taylor [70] reported, the gas sorption technique gives results a minimum of 2–3 times higher than the Blain value. 

The analyzed powder materials were also specified by their physicochemical characteristics and performance, namely pH value, chemical reactivity, solubility, or content of soluble ions. The pH of the leached solution (1:10), which means 90 g of analyzed diatomaceous earth powder was immersed in 900 mL^3^ of distilled water for 24 h, was tested according to EN 12457-2 [71]. The analysis was performed with a pH glass electrode using the potentiometric method. A device inoLab pH/ION 740 (WTW) was used for the pH measurement. Before the measurement, the most important step was to calibrate the pH electrode with corresponding standard pH solutions. The pH values of the tested solutions are presented in Table 7. The different pH values declare the diverse origin of the tested diatomaceous earth that can influence the conditioning of reaction products.

**Figure 4:**
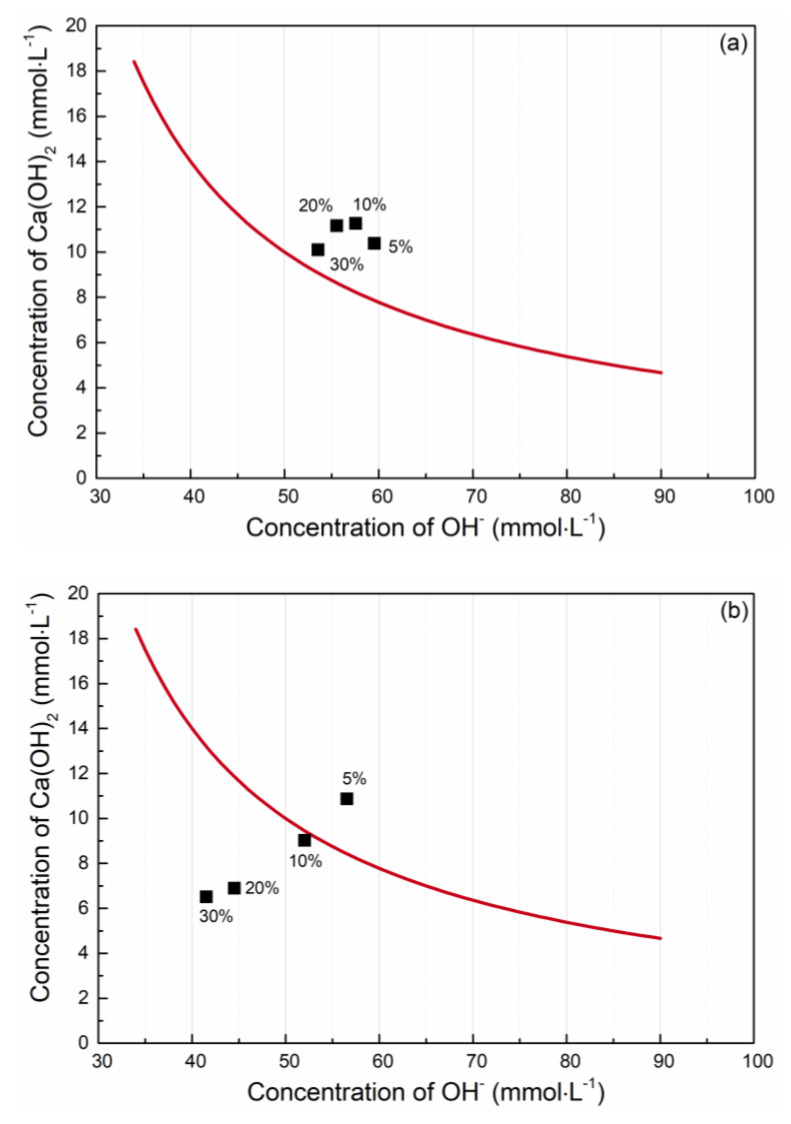

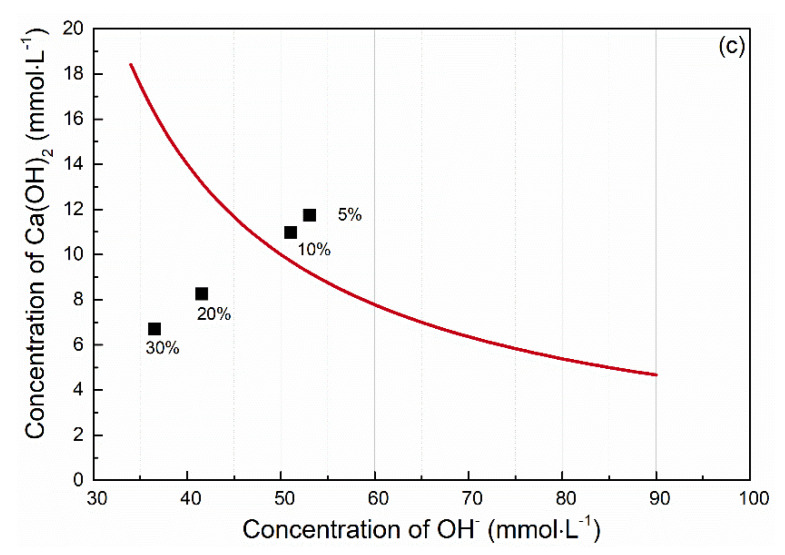
The pozzolana activity analysis of Diatomaceous earth using Frattini test, (**a**) ENO3, (**b**) ENO7, (**c**) BOR.

The pozzolanic activity was determined using the Frattini test, specified in EN 196-5 [72]. The tested blends were prepared using CEM I 42.5 R (Radotín, Czech Republic) that was replaced by 5, 10, 20, and 30 wt.% with the researched diatomaceous earth. The tested cement/diatomite blends were mixed with 100 mL^3^ of boiled distilled water. Then, the samples were put in a sealed plastic bottle and placed in an oven at 40 °C. After 8 days, samples were filtered with a Buchner funnel. The filtrate was firstly analyzed to determine [OH^−^] concentration with titration of HCl using a bromophenol blue indicator. Then, pH was adjusted to 12.5 by NaOH, and titration with EDTA using Murexide indicator [Ca_2^+^_] concentration was assessed. The calculated average values from the three measurements present one point in the pozzolanicity diagram. The test result is satisfactory if the concentration of dissolved Ca(OH)_2_ in suspension is lower than that of the saturated solution. The analyzed sample is pozzolana active as the point lies under the isotherm of Ca(OH)_2_ saturation, which is visible in Figure 4 a–c. Based on the Frattini test and PC substitution ratio, powders ENO7 and BOR were pozzolana active. 

A modified Chapelle test was used to verify the Frattini test based on determining residual Ca(OH)_2_. This method is described in the French standard NF P18-513 [73]. The principle of the method is based on the reaction of 1 g of tested powder material with 2 g of CaO in 250 mL of water at 80 °C for 24 h. The amount of remaining CaO was analyzed using the same titration method as in the case of the Frattini test. The results were expressed in mg Ca(OH)_2_ fixed by the 1g of tested powder and gave straight information about the pozzolanic activity of the tested sample. Raverdy et al. [74] initially proposed that the mineral admixture is pozzolana active if 1 g of the tested sample absorbs more than 650 mg of Ca(OH)_2_. Results of the Chapelle test are summarized in Table 7.

Based on the amount of fixed Ca(OH)_2_, the lowest pozzolanic activity exhibited ENO3. It bonded by 81% and 98.5% less of Ca(OH)_2_ than ENO7 and BOR, respectively. However, all tested silica-based powders exhibited certain reactivity in the presence of hydrated CaO. The chemical reactivity of pozzolanic activity depends mainly on the size of pozzolana particles and the content of active components; it means the amount of amorphous siliceous and aluminous compounds. Jun-Yuan et al. [75] reported that pozzolana active material with a high percentage of the amorphous phase tends to be more reactive than those with a lower percentage of the amorphous phase. This indicates that diatomaceous earth has a smaller particle size and higher amorphous content and has a larger surface area to provide the silica and alumina compounds for higher pozzolanic activity. In this respect, the poor pozzolanic activity of diatomite ENO3 can be assigned to its lowest a-SiO_2_ and high pH content. ENO7 is richer in amorphous content than BOR. However, its Blaine fineness and BET-specific surface are much lower because of the lesser content of fixed Ca(OH)_2_.

### 2.3. Blended Pastes—Preparation and Characterization

The researched mineral admixtures were used as partial cement replacements in the paste mixtures. The dosage of diatomaceous earth was 5, 10, 15, and 20% of cement mass. Due to the high specific surface of diatomaceous earth, the water/binder ratio was 0.5 and was similar for all tested pastes. The consistency of fresh mixtures was studied in accordance with EN 1015-3 [76]. Initial and final setting times were measured by an automatic Vicat apparatus B 26600 (Form Test) in accordance with EN 196-3 [77]. The measurement was conducted in a 40 mm standard mold using a cylindrical needle with a flat tip area of 1 mm^2^. The workability tests were done at a constant laboratory temperature of (23 ± 1) °C. 

Mineral admixtures are usually applied to reduce the overall heat of hydration, particularly the heat release rate, which controls the temperature rise in hardening blended cement paste. The calorimetric curves of tested mixtures were explored using isothermal calorimeter TAM air (TA Instruments) under the constant temperature of 25 °C. The control paste was prepared Portland cement CEMI 42.5 R (Radotín) with a water/cement ratio of 0.5. Diatomaceous earth was used as a partial PC replacement in modified pastes, 10% by cement mass. The used water/binder ratio was maintained, similar to in the case of reference cement paste. 

XRD and IR analyses were conducted to explore the formation of hydration products, and details on these methods are introduced in Section 2.2. In IR analysis, the researched paste mixtures were analyzed in the chosen time intervals which corresponded to the progress of calorimetric curves of the relevant mixture. The hydration process was stopped by rinsing with ethanol on a glass frit filter. 

Properties of the hardened cement pastes were tested on standard 40 mm × 40 mm × 160 mm prisms. The samples were continuously cured in water and tested after 28 days and 90 days, respectively. The measured structural parameters were bulk density, specific density, and total open porosity. These were assessed on dried samples; the samples were thoroughly dried at 60 °C before testing. The dry bulk density *ρ_b_* (kg/m^3^) was obtained using a gravimetric principle in accordance with the standard EN 1015-10 [78]; specific density was measured by the automatic helium pycnometer Pycnomatic ATC (Thermo Scientific). The expanded combined uncertainties of these tests were 1.4% and 1.2%, respectively. The total open porosity *ψ* (%) was calculated from the obtained bulk density and specific density, with the expanded combined uncertainty equaling 2.0%. Flexural *f_f_* (MPa) and compressive *f_c_* (MPa) strength were determined in compliance with EN 1015-11 [79]. A free point bending test was arranged and conducted for flexural strength assessment. Young’s modulus was measured on a dynamic principle using ultrasound working device DIO 562 (Starmans). The uncertainty in determining mechanical parameters was 1.4% for *f_f_* and *f_c_*, and 2.3% for *E_d_*. 

## 3. Results and Discussion

### 3.1. Properties of Fresh Pastes

The values of spreading and data on the initial and final setting time of the analyzed pastes prepared with the constant water/binder ratio of 0.5 are summarized in Table 8. Here, data for pastes with 30% PC replacement are also introduced. 

The highest value of spreading was recorded for reference paste RF. Typically, with the higher dosage of diatomaceous earth in a paste mixture, the spreading dropped, and workability was reduced, whereas the lower values of spreading were recorded for pastes with ENO3. However, the relative differences in spreading were for all three analyzed diatomaceous earth low. The Vicat setting began after 215 min. and 400 min. in the case of ENO7 enriched pastes and reference paste. It was completed after 350 to 690 min for BOR modified pastes and reference paste, respectively. It is apparent that the initial and final setting times significantly depended on the amount of applied diatomaceous admixtures. This occurs well after the sulfate reactions have stopped. However, the C-S-H formation in the acceleration phase of C_3_S dissolution displays the exact time dependence of the setting process [58]. It seems the investigated diatomaceous earth supports the hydration process and plays a micro-filler role. 

**Figure 5:**
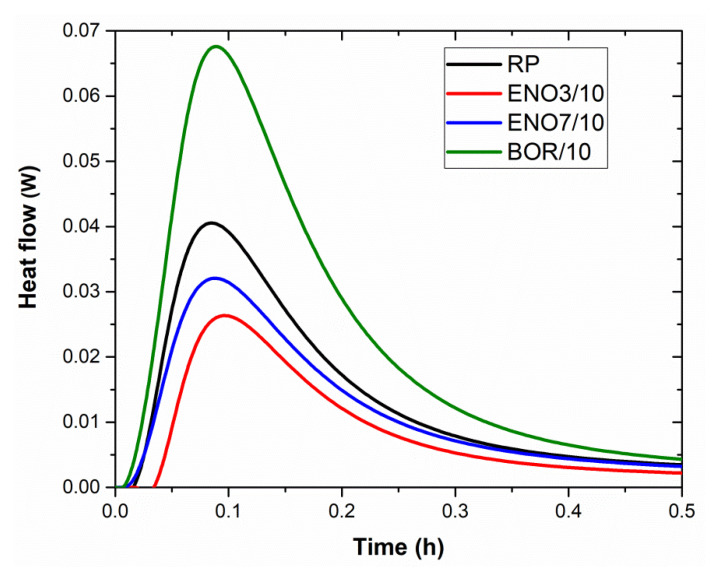
The calorimetric curves of tested pastes were obtained during the first 30 min.

**Figure 6:**
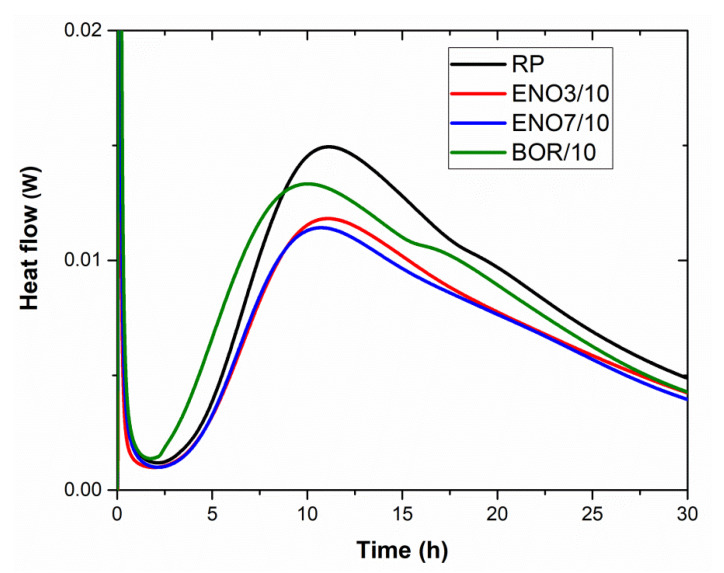
The calorimetric curves of tested pastes were obtained during the first 30 h.

The calorimetric curves of the investigated mixtures are presented in Figure 5 and Figure 6. In the initial phase of hydration, during the first 15 min after mixing with water, the fast development of a high amount of hydration heat took place, which is caused due to hydrolysis and dissolution of ions [80]. It follows a period of relative inactivity, the induction or dormant period. The cement mixture states plastic for several hours, it continues dissolution of ions, and the initial set can be determined. In the case of diatomite BOR, the gypsum reaction is influenced, accompanied by a higher value of hydration heat after one hour of hydration (Table 9) and a shortening of the dormant period, which finished after 1.4 h. The dormant period of other tested pastes ended after approx. 2.3 h from mixing. Then the silicates continued to hydrate rapidly, reaching a maximum rate at the end of the acceleration period, which corresponds with the maximum rate of heat evolution. For reference, cement paste is 11.15 h, for pastes enriched with diatomaceous earth ENO3, ENO7, and BOR, 11.1 h, 10.66 h, and 9.96 h, respectively. By this time, the final set has been passed, and early hardening has begun, corresponding to the values of setting time measured by the Vicat apparatus. The rate of reactions, thereafter, slows down until reaching a steady state. After 24 h, the development of hydration heat of diatomite enriched pastes was comparable with the value of hydration heat of reference paste, except for cement paste with ENO3, whose hydration heat was the lowest (see Table 9). 

The type of phase precipitating during the hydration of blended binder is conditioned by chemistry, initially the hydrolysis/dissolution chemistry of the primary solid binder. Subsequently, the tendency for product growth means product crystallization and polymerization. XRD and IR analyses were conducted to explore the formation of hydration products. The XRD analysis of pastes performed after 1 h of hydration and the formed substances are presented in Table 10.

The primary pozzolanic reaction can be simply described as

(1) CH+S+H→C−S−H

Its kinetics is more similar to the slow rate of C_2_S hydration. The heat of this reaction is about −12 kJ/mole of CH compared to—43 kJ/mole of C_2_S. Thus, the addition of pozzolan (in this case, diatomaceous earth) has a similar effect to raising the C_2_S content of cement, thereby lowering the amount of early heat evolution and reducing early strength, but not long-term strength [80]. This was confirmed for PC blends with ENO3 and ENO7, whose hydration heat and overall heat development were lower and slower than that of reference paste RP. On the other hand, considering the results of heat evolution testing and values of 1-h hydration heat, diatomaceous BOR acted as a highly reactive pozzolan. With very reactive pozzolans content, the C/S ratio is significantly different, being close to 1.0, and the H/S ratio is slightly lower. This can be indicated as the secondary pozzolanic reaction with C-S-H, which can be written as

(2) $$C_{3}S+2S+10.5H\to 3\left[C-S-H_{3.5}\right]$$

As diatomaceous earth BOR contains a significant amount of Al_2_O_3_, which might be potentially reactive, separate secondary reactions occur, which leads to the formation of calcium aluminate hydrates

(3) CH+A+H→C−A−H

**Figure 7:**
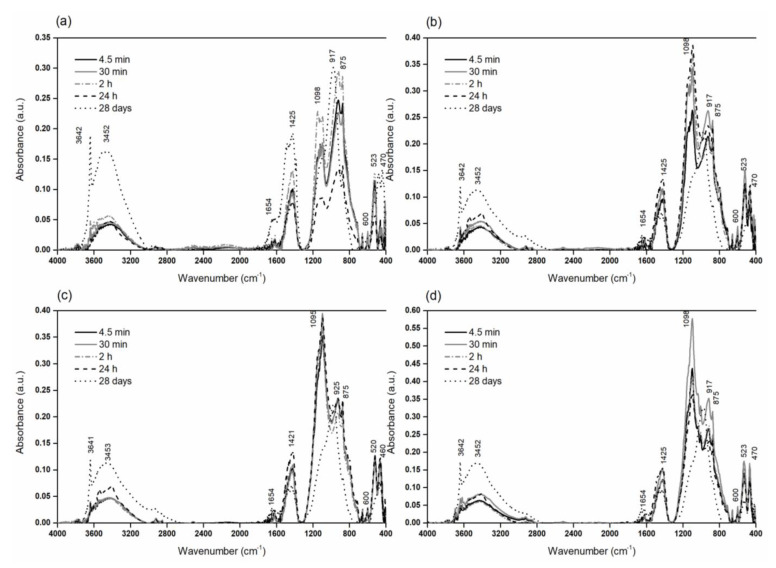
The IR analysis of the hydration process of (**a**) ROR, (**b**) ENO3/10, (**c**) ENO7/10, (**d**) BOR/10.

The hydration process of tested cement pastes with 10% mass replacement of cement with diatomaceous earth was also monitored using IR analysis. The FTIR spectra are graphed in Figure 7, and Table 11 shows the assignment of the major absorption bands of tested mixtures.

The intensity of absorption bands of hydrated mixtures assigned to portlandite (~3641 cm^−^^1^), water (~3500–3400 cm^−^^1^ and ~ 1600–1650 cm^−^^1^), and calcite (1426, 1385, and 875 cm^−^^1^) is increased in comparison with pure Portland cement paste. The maximum absorption of O-H stretching vibration is moved to the higher wavenumbers, evincing more intensive intermolecular water interactions in hydrated compounds. The formation of the C-S-H phase (calcium silicate hydrate) is accompanied by the movement of maximum stretching vibration of SiO_4_^4−^ (925 cm^−^^1^) of hatrurite (Ca_3_SiO_5_) to higher frequencies (968 cm^−^^1^). The movement of stretching vibration of SiO_4_^4-^ (917 cm^−^^1^) to higher frequencies (925 cm^−^^1^) is due to the hydration of larnite (β-Ca_2_SiO^4^). The ettringite formation strongly affects the initial cement hydration [89]. Therefore, it is supposed that the behavior of blended cement pastes might be different at early and later ages. In this respect, longtime monitoring of PC/diatomaceous earth blends will be necessary for future research. The slow rate of pozzolanic reaction requires a prolonged period of moist curing to exploit the full benefits of adding a pozzolan. Without sufficient moist curing, pozzolan will act mainly as a microfiller. 

### 3.2. Properties of 28-Days and 90-Days Pastes

The structural parameters of hardened pastes are summarized in Table 12. Data for 28-days and 90-day samples are presented. Typically, due to the progress in hydration and pozzolanic reactions, the increase in the bulk density values was observed over time which was further reflected in the lowered porosity. The examined mineral admixtures affected the structure of the hardened pastes differently. For ENO3, the drop in porosity exhibited pastes ENO3/5 and ENO3/10. For mixtures with a higher dosage of diatomaceous earth was, the porosity slightly higher than that of reference paste, and the redundant mineral admixture served as filler only. ENO7 decreased the porosity of blended pastes for PC replacement up to 15 wt.%. Contrary to that, using diatomaceous earth, BOR oppositely affected the porosity. The porosity was slightly increased for cement replacement ratio >15 wt.%. However, in this case, the diatomaceous earth also contributed to the cement matrix’s densification, which was well apparent for pastes BOR/5 ad BOR/15. The porosity results can be assigned to the increased fraction of CSH that provides a more homogeneous and compact microstructure, particularly if highly reactive, finely powdered pozzolans are used. In evaluating structural parameters, one must consider the differences in the progress of hydration and pozzolanic reactions of blended pastes and reference PC paste. 

The results of the mechanical parameters assessment and values of strength activity index (SAI) are presented in Table 13. SAI was determined as a ratio of 28-days and 90-day compressive strength of the chosen blended paste and that of reference paste RP [90]. Similarly, as in the case of structural parameters, the prolonged curing time led to the densifying of studied pastes and, thus, an increase in the examined mechanical parameters. For 28-day samples, the lowest SAI (92.9%) was obtained for BOR/20 and the highest (113.6%) for ENO7/10. The 90-days SAI varied from 88.9% to 109.1%. The results of the SAI assessment gave clear evidence of pozzolanic reactivity of researched mineral admixtures. As SAI of 75% is usually considered as limiting value to classify mineral admixture among pozzolans, this criterion was safely met for all analyzed diatomaceous earth and cement blends. For ENO3, prolonged hydration was detected. Best mechanical performance yielded blended paste with ENO7, which proved its high reactivity. For all studied ENO7-enriched pastes, the compressive strength was higher or similar to that of reference paste except for paste with 20% cement substitution. In the case of ENO3, the drop in mechanical parameters was recorded for diatomaceous earth dosage >10 wt.%, but SAI was still satisfactory to rank this material as pozzolana active. The results of mechanical parameters testing agree with those reported by Değimenci and Yilmaz [51]. They reported that the compressive strength of mortars containing diatomite increases with age, and the rate of increase depends upon the cement replacement level. Similarly, Jang-Hyun and Chang-Bok [91] confirmed that diatomite could be used as an admixture to reinforce concrete durability and increase strength efficiency. 

## 4. Conclusions

The following main conclusions can be drawn from the conducted tests and analyzes:

(i) Three basic factors influence the contribution of diatomaceous earth to cement mixture strength when partially replacing Portland cement. These are the filler effect, the pertinent acceleration of ordinary Portland cement hydration, and the pozzolanic reaction of diatomite with cement hydrates.
(ii) The filler effect is immediate. The acceleration of ordinary Portland cement hydration has a significant impact within the first 24 h, leading to the shortening of both initial and final setting time.
(iii) Due to the chemical and phase composition, particle size, and morphology of diatomaceous particles, the examined blended pastes exhibited distinct rheology, heat release, the time evolution of hydration and pozzolanic reactions, structural parameters, and, consequently, mechanical strength.
(iv) The progress in hydration and pozzolanic reactions was apparent from assessing 28-day and 90-day hardened samples.
(v) Based on received SAI values, the researched mineral admixtures were classified as highly reactive pozzolans, which can be beneficially applied in the design and production of potentially more durable repair mortars applicable also for historical masonry with excessive moisture presence. However, the analyzed diatomaceous earth can also find use in the production of blended cement, cement-based composites, or as a part of multi-component binders. The ability to improve the reactivity of diatomite by additional grinding must also be considered in the preparation of blended binders.
(vi) The optimum replacement level of ordinary Portland cement by diatomaceous earth to give maximum long-term strength enhancement is about 10 wt.%. However, an even higher dosage of diatomaceous earth provides materials of sufficient strength and Young’s modulus. For example, in the case of ENO7, the improvement in mechanical parameters was found to be up to 15% Portland cement replacement. With respect to identified SAI, the safe value of Portland cement substitution is 20 wt.%.

## Figures and Tables

**Table 1:** Chemical composition of base materials.

Parameter, Substance	CEM I 42.5 R	ENO3	ENO7	BOR
	Content (Mass %)			
Annealing loss at 105 °C	0.40	0.05	82.58	3.30
Insoluble fraction	1.63	2.00	1.20	2.48
SiO_2_	19.00	89.70	90.90	82.90
Al_2_O_3_	4.31	3.40	3.20	13.20
Fe_2_O_3_	2.40	2.10	3.10	1.50
TiO_2_	0.28	0.60	0.60	0.60
CaO	62.90	0.60	0.57	0.30
MgO	1.80	0.20	0.20	0.20
K_2_O	0.82	0.30	0.30	0.90
Na_2_O	0.14	2.70	0.20	0.10
P_2_O_5_	0.16	0.10	0.80	0.10
SO_3_	3.24	0.00	0.00	0.10

**Table 2:** XRD data of diatomites.

Substance	ENO3	ENO7	BOR
	Content (wt.%)		
amorphous phase	37.70	66.10	53.90
Opal CT	22.05	0.00	0.00
SiO_2_ quartz	0.00	17.00	6.00
SiO_2_ tridymit	1.10	0.00	0.00
SiO_2_ cristobalite	39.20	16.10	0.00
Al_2_(Si_2_O_5_)(OH)_4_ Kaolinite	0.00	0.00	32.70
K_2_(Al_4_FeO)(Si_6_Al_2_O_20_)(OH)_4_ illite	0.00	0.00	7.30

**Table 3:** Assignments of the major absorption bands of CEM I 42.5 R, Radotín [57].

Wavenumbers (cm^−1^)	Assignment
3641	ν (O-H) in portlandite (Ca(OH)_2_)
3552	ν (O-H) in water, chemisorption of water on the surface of molecules
3420	ν (H-O-H) in water, chemisorption of water on the surface of molecules
2513, 1426, 876, 715	ν (C=O) in calcite (CaCO_3_)
1617	ν_2_ (O-H) in water
1384 1152, 1114, 1095	ν (C=O) in CO_3_^2−^ compounds ν_3_ (SO_4_^2−^) in gypsum, basanite, and anhydrite [58]
970, 924	ν_3_ (Si-O) in C_3_S
902, 780, 516, 509, 421	ν (Al-O) in C_3_A
845	ν_4_ (Si-O) in β−C_2_S
522	ν_4_ (Si-O) in C_3_S
700–500 459	ν (Fe-O) in C_4_AF ν_4_ (Si-O-Si)

**Table 4:** Assignments of the major absorption bands of tested diatomaceous earth [59].

Wavenumbers (cm^−1^)	Assignment
3696 3651 3620	ν (H-O-H) free ν (O-H) inner surface [60] ν (O-H) in crystalline molecules
~3436 1631	ν (H-O-H) in water, chemisorption of water on the surface of molecules ν (H-O-H) in water in minerals
1385	ν (C=O) in CO_3_^2−^ compounds
1099	ν (Si-O-Si) asymmetric in plane vibration
950 797, 695 650–500 538 470	ν (Si-OH) ν (Si-O) ν (Si-O-Al^IV^) ν (Fe-O) in Fe_2_O_3_ ν_4_ (Si-O-Si)

**Table 5:** Particle size distribution parameters.

Material	Size (µm) Freq d_10_	Size (µm) Freq d_50_	Size (µm) Freq d_90_
CEMI 42.5R	5.8	23.4	35.5
ENO3	22.3	37.5	63.5
ENO7	19.6	29.0	43.0
BOR	9.5	23.0	32.5

**Table 6:** Physical properties of cement and diatomaceous earth.

Material	Blaine Fineness (m^2^·kg^−1^)	BET Specific Surface Area (m^2^·kg^−1^)	Specific Density (kg·m^−3^)
CEMI 42.5R	360	5 500	2 860
ENO3	785	2 494	2 334
ENO7	1 865	2 007	2 399
BOR	2 095	18 312	2 416

**Table 7:** Physico-chemical properties of tested materials.

Material	pH Value (-)	Pozzolanic Activity (mg Ca(OH)_2_ Fixed by 1 g)
CEM I 42.5R	12.45	1147
ENO3	9.34	246
ENO7	7.11	1349
BOR	6.49	1614

**Table 8:** The values of spreading and setting time of tested pastes.

Paste Mixture	Value of Spreading (mm)	Initial Setting Time (min.)	Final Setting Time (min.)
RP	>300	400	690
ENO3/5	280 × 280	355	635
ENO3/10	240 × 245	330	635
ENO3/15	210 × 210	280	500
ENO3/20	180 × 180	245	375
ENO3/30	150 × 145	240	350
ENO7/5	255 × 260	380	680
ENO7/10	210 × 210	315	640
ENO7/15	175 × 175	225	410
ENO7/20	140 × 140	215	395
ENO7/30	140 × 140	215	390
BOR/5	260 × 260	375	670
BOR/10	210 × 210	350	640
BOR/15	165 × 165	250	450
BOR/20	145 × 145	240	355
BOR/30	140 × 140	220	350

**Table 9:** Hydration heat of tested pastes relative to cement weight.

Paste Mixture	1 h (J·g^−1^)	24 h (J·g^−1^)
RP	7.56	203.15
ENO3/10	1.04	161.87
ENO7/10	1.63	202.08
BOR/10	11.77	202.08

**Table 10:** Results of XRD analysis of cement pastes after 1 h of hydration.

Substance	ENO3/10	ENO7/10	BOR/10
	Content (Mass %)		
Amorphous phase	70.00	65.90	66.90
Aluminum oxide chloride	0.10	0.30	0.30
Cristobalite	1.30	0.90	-
Quartz	0.60	1.30	1.00
Hatrurit	5.40	3.90	4.60
Portlandite	13.00	15.80	16.90
Ettringite	1.00	2.10	1.10
Calcium carbonate	8.60	10.00	9.30

**Table 11:** Assignments of the major absorption bands of hydrated mixtures.

Wavenumber (cm^−1^)	Assignment
3641, 3642	ν (O-H) crystalline hydroxyl [81], e.g., portlandite (Ca(OH)_2_) [82]
3424–3553	ν (H-O-H) in liquid water, chemisorption of water on the surface of molecules [83]
1617–1653	ν_2_ (H-O-H) in water [84]
1426, 1384, 875–873	ν (C=O) in CO_3_^2−^ compounds, calcite [85]
1095–1098	ν_3_ (SO_4_^2−^) ettringite
925–968	ν_3_ (Si-O) in the C-S-H phase [86]
917–925	ν_4_ (Si-O) in the C-S-H phase [86]
780–918	δ (O-H) linked to Al^3+^ [81]
599–601	ν (Al-O) in C_3_A [85]
520–523	ν_4_ (Si-O) in C_3_S, β-C_2_S, C_3_A [87]
700–500	ν (Fe-O, Al-O) in C_4_AF [88]
440–470	ν_4_ (Si-O-Si) in the C-S-H phase [87]

**Table 12:** The basic material properties of tested mixtures.

Paste	Bulk Density (kg·m^−3^)		Specific Density (kg·m^−3^)		Open Porosity (%)	
	28 Days	90 Days	28 Days	90 Days	28 Days	90 Days
RP	1558 ± 22	1589 ± 22	2181 ± 26	2159 ± 26	28.6 ± 0.6	26.4 ± 0.5
ENO3/5	1451 ± 20	1495 ± 21	2017 ± 24	2008 ± 24	28.1 ± 0.6	25.5 ± 0.5
ENO3/10	1422 ± 20	1460 ± 20	2007 ± 24	1991 ± 24	29.1 ± 0.6	26.7 ± 0.5
ENO3/15	1375 ± 19	1416 ± 20	1966 ± 24	1951 ± 23	30.1 ± 0.6	27.4 ± 0.5
ENO3/20	1319 ± 19	1364 ± 19	1925 ± 23	1907 ± 23	31.5 ± 0.6	28.5 ± 0.6
ENO7/5	1576 ± 22	1594 ± 22	2154 ± 26	2141 ± 26	26.8 ± 0.5	25.5 ± 0.5
ENO7/10	1610 ± 23	1623 ± 23	2163 ± 26	2149 ± 26	25.6 ± 0.5	24.4 ± 0.5
ENO7/15	1549 ± 22	1573 ± 22	2148 ± 26	2132 ± 26	27.9 ± 0.6	26.3 ± 0.5
ENO7/20	1473 ± 21	1488 ± 21	2083 ± 25	2069 ± 25	29.3 ± 0.6	28.1 ± 0.6
BOR/5	1492 ± 21	1517 ± 21	2078 ± 25	2072 ± 25	28.2 ± 0.6	26.8 ± 0.5
BOR/10	1434 ± 20.1	1456 ± 20	2021 ± 24	2008 ± 24	28.9 ± 0.6	27.5 ± 0.6
BOR/15	1411 ± 19.8	1425 ± 20	1996 ± 24	1981 ± 24	29.3 ± 0.6	28.1 ± 0.6
BOR/20	1349 ± 18.9	1371 ± 19	1936 ± 23	1922 ± 23	30.3 ± 0.6	28.7 ± 0.6

**Table 13:** Mechanical parameters of hardened pastes.

Paste	Flexural Strength (MPa)		Compressive Strength (MPa)		SAI (%)		Young’s Modulus (GPa)	
	Days							
	28	90	28	90	28	90	28	90
RP	7.3 ± 0.1	8.2 ± 0.1	52.3 ± 0.7	58.4 ± 0.8	-	-	14.5 ± 0.2	16.7 ± 0.2
ENO3/5	7.5 ± 0.1	8.7 ± 0.1	52.5 ± 0.7	59.2 ± 0.8	100.4	101.3	14.5 ± 0.2	17.1 ± 0.2
ENO3/10	7.2 ± 0.1	8.4 ± 0.1	50.0 ± 0.7	58.1 ± 0.8	95.6	99.5	14.2 ± 0.2	16.7 ± 0.2
ENO3/15	6.6 ± 0.1	7.8 ± 0.1	48.2 ± 0.7	54.7 ± 0.8	92.1	93.7	13.6 ± 0.2	15.1 ± 0.2
ENO3/20	6.0 ± 0.1	7.3 ± 0.1	43.1 ± 0.6	53.0 ± 0.7	82.4	90.8	12.1 ± 0.2	14.1 ± 0.2
ENO7/5	7.8 ± 0.1	9.0 ± 0.1	55.8 ± 0.8	60.6 ± 0.8	106.7	103.8	15.8 ± 0.2	17.2 ± 0.2
ENO7/10	8.4 ± 0.1	9.4 ± 0.1	59.4 ± 0.8	63.7 ± 0.9	113.6	109.1	16.8 ± 0.2	17.4 ± 0.2
ENO7/15	8.0 ± 0.1	8.5 ± 0.1	54.4 ± 0.8	58.4 ± 0.8	104.0	100.0	15.9 ± 0.2	17.0 ± 0.2
ENO7/20	6.9 ± 0.1	7.7 ± 0.1	50.2 ± 0.7	53.8 ± 0.8	96.0	92.1	14.0 ± 0.2	14.7 ± 0.2
BOR/5	7.6 ± 0.1	8.5 ± 0.1	52.6 ± 0.7	59.4 ± 0.8	100.6	101.7	14.9 ± 0.2	17.1 ± 0.2
BOR/10	7.4 ± 0.1	8.3 ± 0.1	52.3 ± 0.7	58.9 ± 0.8	100.0	100.9	14.7± 0.2	16.9 ± 0.2
BOR/15	7.0 ± 0.1	7.8 ± 0.1	50.4 ± 0.7	53.1 ± 0.7	96.4	90.9	14.3 ± 0.2	14.7 ± 0.2
BOR/20	6.7 ± 0.1	7.1 ± 0.1	48.6 ± 0.7	51.9 ± 0.7	92.9	88.9	13.70 ± 0.2	13.7 ± 0.2

## Data Availability

The data presented in this study are available on request from the corresponding author. The data are not publicly available due to privacy.
